# Internal Hernia: A Rare Cause of Bowel Ischemia and Infarction

**DOI:** 10.7759/cureus.78482

**Published:** 2025-02-04

**Authors:** Banwari Lal Bairwa, Safiyyah Manjra, Shubham Gupta

**Affiliations:** 1 Surgery, MP Birla Hospital and Research Center, Chittorgarh, IND; 2 Medicine and Surgery, The University of the West Indies, Bridgetown, BRB; 3 Surgery, Dr. D. Y. Patil Medical College, Kolhapur, IND

**Keywords:** bowel ischemia, congenital internal hernia, exploratory laporotomy, intestinal obstruction, ischemic infarction, left paraduodenal hernia, surgical acute abdomen

## Abstract

Internal abdominal hernias are uncommon causes of intestinal obstruction and are difficult to detect preoperatively due to their imprecise clinical manifestations and complicated anatomic appearance.

Paraduodenal hernias represent the most common subset of all internal hernias (IHs). The symptoms are nonspecific and range from persistent abdominal discomfort to acute abdomen. An expert radiologist and a high index of suspicion of IH are required to diagnose timely and to prevent its fatal complications, such as bowel ischemia and infarction. An IH is a potentially fatal entity if misdiagnosed and not treated timely.

We present a case of a 16-year-old boy managed with an exploratory laparotomy for a strangulated internal (left paraduodenal) hernia in an emergency. A total of 274 cm of small bowel was resected, and only 140 cm of jejunum was left behind. A jejunostomy was created. This case report reveals the importance of timely management and fatal complications due to delayed diagnosis and treatment of IHs.

## Introduction

Acute abdomen due to intestinal obstruction is the most common surgical emergency with a lot of underlying causes. Intestinal obstruction due to internal hernias (IHs) accounts for 0.6% to 5.8% of all small bowel obstructions [1]. IH forms when there is a protrusion of intra-abdominal contents, most often small bowel, through a normal or abnormal peritoneal or mesenteric defect [1,2].

Paraduodenal hernias (PDHs) are rare types of hernias that result from incomplete rotation of the midgut [2]. Paraduodenal, or Treitz hernias, are the most frequent type of IH caused by midgut rotational abnormalities during embryogenesis. The left paraduodenal fossa (Landzert fossa) hernia is the most prevalent (75%), followed by the right paraduodenal fossa (Waldeyer fossa) hernia (25%). The incidence of left PDH is higher than right PDH and the incidence ratio of left PDH to right PDH is about 3:1. The incidence of PDH is higher in males than females [1,2]. It is difficult to diagnose because of its undefined clinical characteristics, similar to other intestinal obstructions like abdominal pain and distension, vomiting, obstipation, etc.

If an IH is misdiagnosed or left untreated, it can result in strangulation or incarceration of the bowel, resulting in higher rates of morbidity and mortality in patients [2,3]. It is rarely found in adults and has been reported as a cause of small bowel obstruction in 0.6-5.8% of cases [3]. Incarcerated IHs with symptomatic presentation are a surgical emergency, and if not reduced early, can lead to strangulation and further progress to ischemia, infarction, or perforation of the bowel [4,5], and mortality rates of up to 45% have been reported [4]. The gangrenous or ischemic small bowel requires surgical excision of the bowel, which can result in small bowel syndrome [6]. Prompt surgical intervention is based on the clinical picture of the patient and/or findings on computed tomography (CT) scans assist in better management of the patient with an IH [3]. We describe here a rare case of IH presenting with ischemia of 9 feet of small bowel in a boy.

## Case presentation

A 16-year-old boy presented in the emergency department with severe abdominal pain, vomiting, and abdominal distension with a history of the same complaint for the last three days. He consulted with a local practitioner but was not relieved, so he referred him to us for further management. There was no history of similar illness or any surgery in the past.

At presentation, blood pressure was 96/58 mm Hg, pulse rate was 112/min, SpO_2_ on air was 98%, and the patient was moderately dehydrated. On abdominal examination, generalized tenderness with guarding and rigidity was present. Sluggish bowel sounds were audible on auscultation. Blood investigations revealed a white blood cell count of 16.5 × 10^9^/L, a neutrophil percentage of 92%, a hemoglobin of 11.5 G/L, a C-reactive protein concentration of 284 mg/L, and a serum lactate concentration of 1.9 mmol/L.

**Figure 1:**
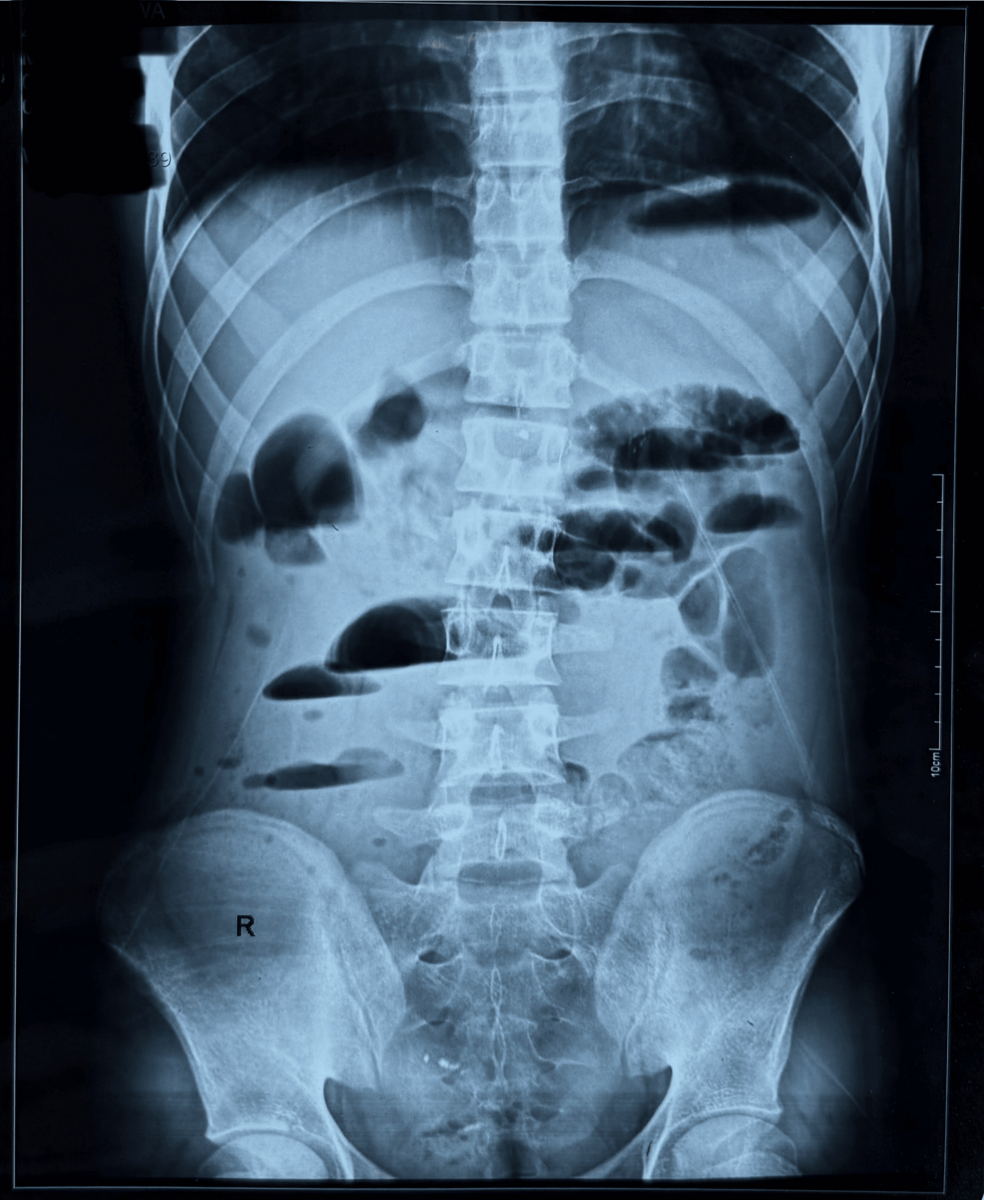
Abdominal radiograph showed dilated intestinal loops and multiple air-fluid levels.

The abdominal radiograph showed dilated bowel loops left lateral to the midline and multiple air-fluid levels (Figure 1).

Abdominal ultrasonography revealed dilated small bowel loops. A CT scan was not performed due to the patient being hemodynamically unstable, and a diagnosis of acute abdomen was made. After resuscitation, the patient was taken for emergency exploratory laparotomy.

**Figure 2:**
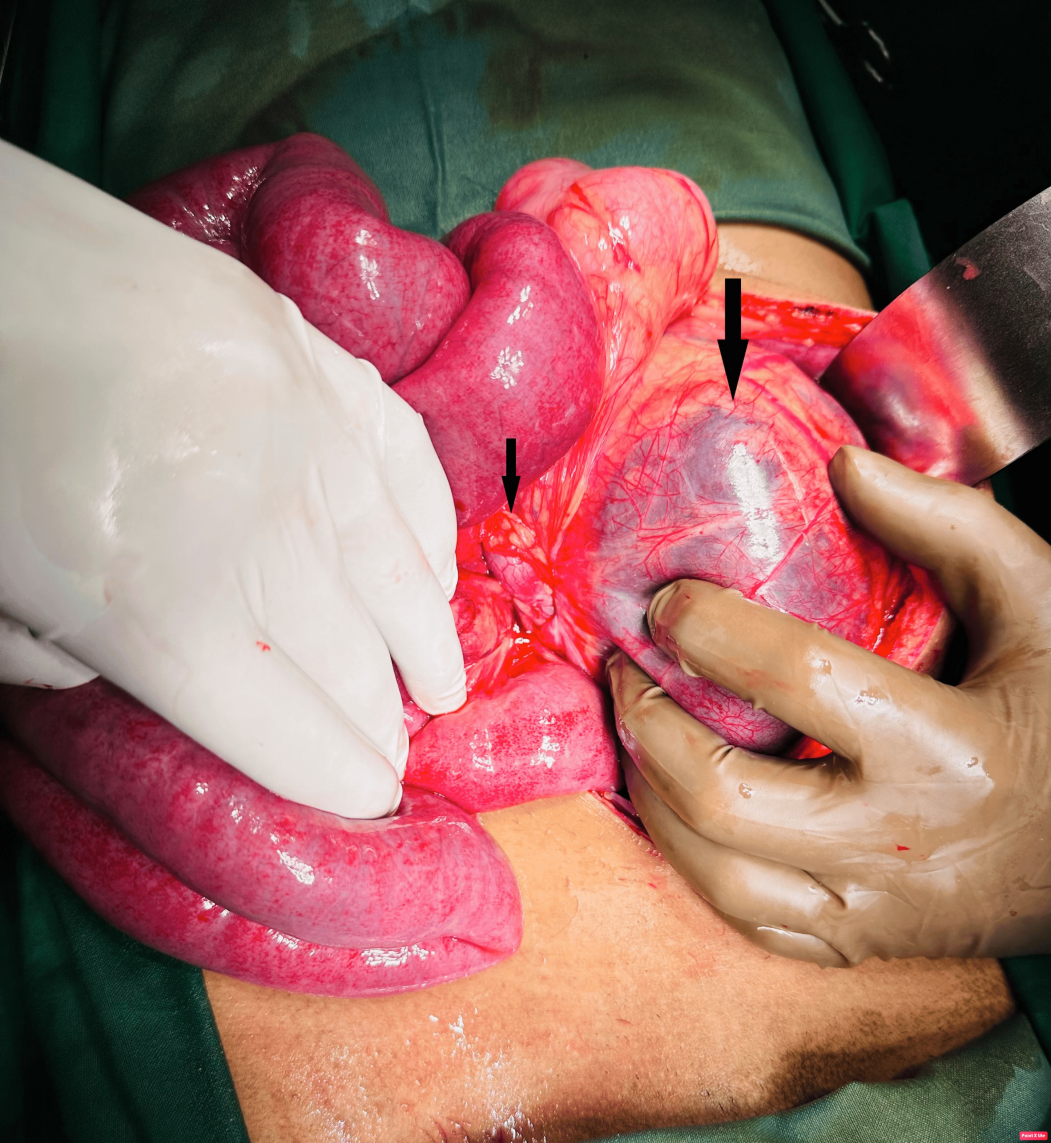
Intraoperative image showing a cluster of small bowel loops in the hernia sac and neck of the hernia sac.

An exploratory laparotomy with a midline incision was performed. On exploration, we found a mesenteric sac on the left side of the abdomen containing strangulated and ischemic small bowel loops (Figure 2).

**Figure 3:**
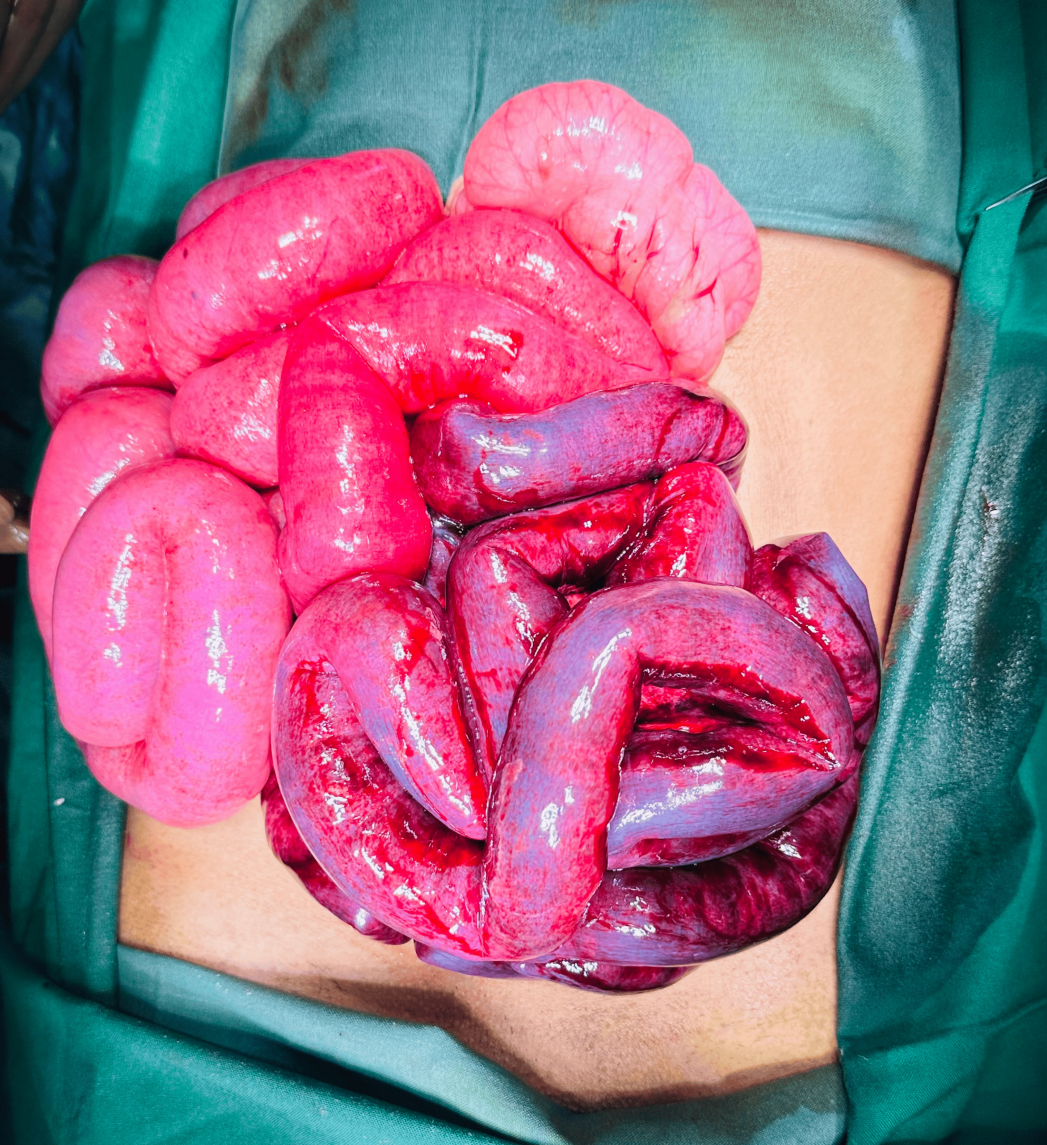
Intraoperative ischemic appearance of a large part of the small bowel after the reduction from the sac.

A defect was noted in the paraduodenal region in the Landzert foramen, with a majority of the small bowel in the hernia sac. The sac was opened, and gangrenous bowel was delivered, and some amount of bloody exudate was present in the sac (Figure 3).

**Figure 4:**
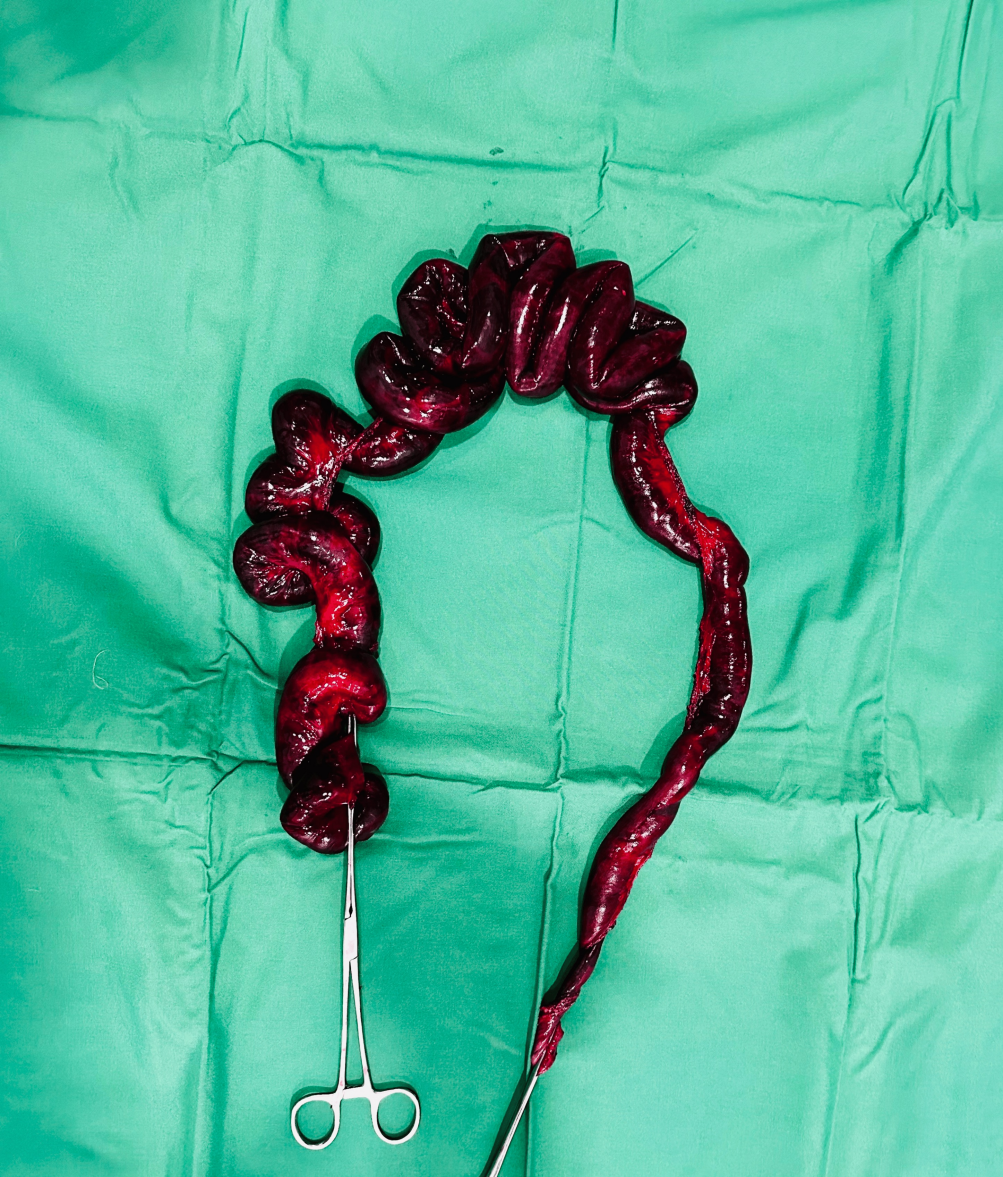
Resected large segment of gangrenous small bowel.

The hernia sac neck was released, and the herniated small bowel was removed. The hernia defect and the mesenteric defect were closed. Ischemic changes were present around 9 feet of the small bowel. Hot fomentation of the bowel was done for 10-15 minutes, and the viability of the bowel was checked. Approximately 9 feet of small bowel from 5 feet distal to the duodenojejunal junction to the ileocolic junction was ischemic and necrosed. An ischemic large segment of the small bowel was resected (Figure 4). No colonic resection was done.

Due to inflammation and edema of the bowel, primary anastomosis was not done, and jejunostomy was created without a mucus fistula. Postoperatively, the patient was managed in the ICU with intravenous fluids, antibiotics, and total parenteral nutrition. The patient was discharged on postoperative day 8 with a jejunostomy and on a diet as advised by a dietitian.

**Figure 5:**
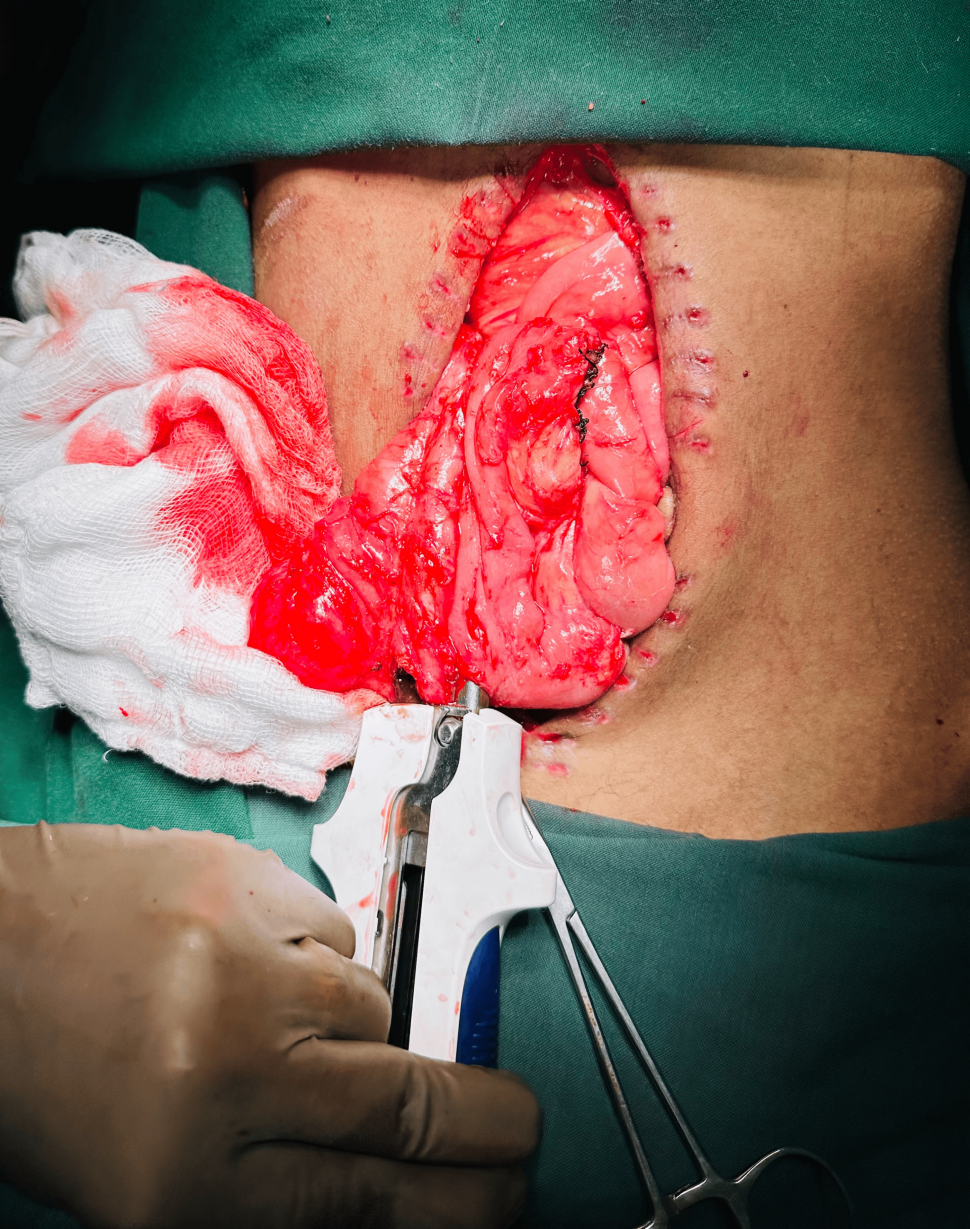
Intraoperative images showing jejuno-colic stapler anastomosis.

One month later, the patient was planned for jejunostomy reversal. Re-exploration was done. Jejunostomy was mobilized, and side-to-side jejunocecal anastomosis was done successfully (Figure 5).

A pelvic drain was placed. The midline incision was closed in layers, and the jejunostomy was matured. The postoperative period was uneventful, and the patient was discharged on postoperative day 10 in good condition. At six months of follow-up, the patient is doing well.

## Discussion

IHs are rare congenital abnormalities that can be asymptomatic or present as intestinal obstruction and some potentially life-threatening complications like bowel gangrene and perforation. The incidence of IHs after surgical procedures such as roux-en-Y gastric bypass is up to 16% of cases. IHs have an incidence of 0.2-0.9% among all cases of bowel obstruction [7,8]. PDH is the most common type of IH and approximately 45% of all IHs. Moynihan’s theory suggests that "physiological adhesions" occur when the bowel returns back to the abdomen and the common dorsal mesentery fuses with the posterior abdominal wall, resulting in fusion folds and fossa formation. These fossae gradually increase in size, leading to PDH [9]. The left para-duodenal hernia occurs due to the herniation of intestinal loops into this potential space known as the Landzert fossa, which failed to obliterate [1,9].

Males have a higher incidence of IH than females. IH can be symptomatic or asymptomatic. IH remains a diagnostic challenge due to nonspecific and highly variable clinical symptoms ranging from vague digestive symptoms to features of acute abdomen [10,11]. Most of the patients present with features of intestinal obstruction (abdominal pain, distension, vomiting, and obstipation), and the most frequently herniated organ is the small bowel [11]. The severity of symptoms depends on the duration of obstruction, presence or absence of incarceration, and strangulation. Acute abdomen is usually seen with bowel ischemia, infarction, and perforation later on. Non-specific clinical presentation leads to diagnostic delay, and this results in increased rates of bowel ischemia, gangrene, perforation, and bowel resection. Mortality in patients with complicated IHs ranges from 20% to 50% in the published literature. Early diagnosis and surgical interventions can prevent the patient from having fatal complications [12].

Abdominal radiographs and ultrasonography are poor in detecting the etiology of obstruction, but CT plays a significant role in the diagnosis and management of IHs. CT shows a swirl sign (twisted mesenteric vessels and loop of small bowel). This is also known as the whirlpool sign. It indicates mesenteric volvulus, IH, and closed-loop obstruction [3]. Contrast-enhanced computed tomography (CECT) of the abdomen has an accuracy of 90-95% and a sensitivity of 94.5-100% [12-14]. An experienced radiologist and a high index of suspicion are required to establish the diagnosis preoperatively.

Timely surgical correction is the optimal treatment of IHs because the risk of incarceration and strangulation in untreated cases is 50% [15]. Treatment includes emergency surgery, and the favorable outcome depends on timely, appropriate surgical management. Reductions of the strangulated bowel loops should be done as early as possible to prevent bowel ischemia and infarction [9]. Hernia defects should be closed with a non-absorbable suture to prevent recurrences. Bowel segment resection depends on the viability of the bowel [2,16]. In complicated IHs, exploratory laparotomy is the opted modality of intervention. Our patient was managed by an emergency exploration, and the resection of the small bowel with jejunostomy was done.

## Conclusions

Preoperative diagnosis of IH is challenging. Delays in diagnosis and management can have fatal consequences like bowel ischemia and gangrene. Early diagnosis and timely surgical intervention have a crucial role in the management of IH. Due to nonspecific clinical features, the rate of preoperative diagnosis of IH is low. With a high index of suspicion and quick and timely surgical management, favorable outcomes can be achieved and fatal complications can be avoided. In the absence of previous surgical history, the IH should be included in the differential diagnosis of intestinal obstruction. Contrast-enhanced CT scan remains the gold standard imaging modality. Surgery is the only treatment for symptomatic and complicated IHs.
